# Meta-Analysis of Shrinkage Mode After Neoadjuvant Chemotherapy for Breast Cancers: Association With Hormonal Receptor

**DOI:** 10.3389/fonc.2021.617167

**Published:** 2022-04-04

**Authors:** Chun-Hui Zheng, Kai Xu, Wen-Ping Shan, Ya-Kun Zhang, Zhi-De Su, Xiang-Jin Gao, Yu-Jue Wang, Jian-Yu Qi, Xiao-Yan Ding, Chun-Ping Wang, Yong-sheng Wang

**Affiliations:** ^1^Breast Cancer Center, Shandong Cancer Hospital and Institute, Shandong First Medical University and Shandong Academy of Medical Sciences, Jinan, China; ^2^Department of Breast Surgery, Weifang People’s Hospital, Weifang, China; ^3^Department of Preventive Medicine, Weifang Medical University, Weifang, China; ^4^Department of Radiology and Environmental Medicine, China Institute for Radiation Protection, Taiyuan, China; ^5^Department of Anesthesiology, Weifang People’s Hospital, Weifang, China; ^6^Department of Pharmacy, Weifang People’s Hospital, Weifang, China; ^7^School of Dentistry, University of California Los Angeles, Los Angeles, CA, United States; ^8^Department of Laboratory Medicine, Key Laboratory of Clinical Laboratory Diagnostics in Universities of Shandong, Weifang Medical University, Weifang, China

**Keywords:** breast cancer, neoadjuvant chemotherapy, shrinkage mode, meta-analysis, receptor status

## Abstract

**Background:**

Patients with concentric shrinkage mode after neoadjuvant chemotherapy (NAC) is considered to be ideal candidates for breast conserving treatment (BCT). While, what proportion of patients would represent CSM have not been well defined. This study was conducted to pool the rates of concentric shrinkage mode (CSM) in patients undergoing NAC, determine the impact of hormonal receptor on the shrinkage mode after NAC and estimate the rates of the CSM in various subgroups.

**Methods:**

We conducted a systematic review following the guidelines for Meta-Analyses and Systematic reviews for the PRISMA guidelines. We systematically searched the literature about shrinkage mode after NAC from PubMed, Web of Science, Embase, The Cochrane Library, CNKI, Wanfang database published from January 2002 to June 2020 on breast cancer shrinkage mode after NAC and carefully screened the literature by using eligibility criteria: (1) patients with primary breast cancer treated with NAC; (2) publications with available data of shrinkage mode measured by magnetic resonance imaging (MRI), or data of pathology and hormonal receptor. The association between shrinkage mode and hormonal receptor was estimated using Stata 15.1 software.

**Results:**

This analysis included a total of 2434 tumors from 23 papers. The included studies were heterogeneous (I2 = 89.4%, P<0.01). Random effects model was used to estimate the overall rates of CSM: 56.6% [95%CI (50.5%, 62.7%)]. According to the analysis of hormonal receptor, 10 of the paper was included for HR+ (hormone receptor positive) type analysis and the rate of CSM for HR+ type was 45.7% [95%CI (36.4%, 55.0%)]; 9 of the paper was used for HR- type (hormone receptor negative) analysis and the incidence of HR-CSM is 63.1% [95%CI (50.0%, 76.1%)]; with HR+ type as the control, the OR of the HR- CSM rate is 2.32 (1.32, 4.08) folds of HR+ type. From subgroup analyses, the CSM% of luminal A, luminal B, Her2+, and triple negative were 29.7% (16.5%, 42.8%); 47.2% (19.1%, 75.3%); 59.0% (39.7%, 78.3%); 66.2% (52.8%, 79.6%), respectively.

**Conclusions:**

Breast cancer patients undergoing NAC did not get an ideal odds ratio of CSM. The incidence of CSM in breast cancer after NAC is associated with hormonal receptor. Patients with triple-negative breast cancers have the highest rates of CSM after NAC. More care should be taken to select patients with the luminal subtypes for BCT throughout NAC.

## Introduction

Neo-adjuvant chemotherapy (NAC) is being increasingly used in advanced breast cancers, which could improve the success rate of breast conserving treatment (BCT) by reducing the extent of surgery (1). Previous study reported that the overall proportion of BCT significantly increased from 15.7% to 26.0% from 2010 to 2015 (2). However, tumors downsized by NAC were reported to have higher local recurrence after BCT than those who have not (3). Strategies to decrease the local recurrence after BCT associated with NAC should be made; for example, the shrinkage mode should be assessed accurately beforehand, especially the non-concentric shrinkage mode (NCSM), which can lead to false negative reporting of margins. Therefore, at the St. Gallen international expert consensus conference on the primary therapy of early breast cancer 2017, the experts voted that different surgical strategies should be adopted for breast cancer based on shrinkage mode (4).

Previous studies have shown an association between the shrinkage mode and molecular subtypes (5). However, there are some limitations in these studies, such as a relatively small study population and diverse classification standard. Furthermore, the incidence of concentric shrinkage mode (CSM) in breast cancer with diverse hormonal receptor have not been pooled analysis.

Therefore, we conducted a meta-analysis to estimate the overall proportion of subjects showing CSM after NAC and to determine whether hormonal receptor was associated with shrinkage mode. Therefore, we can understand the clinical benefit from NAC and lead to further individualization of breast cancer surgical management.

## Methods

We conducted a systematic review following the guidelines for Meta-Analyses and Systematic reviews for the PRISMA guidelines (6). Shrinkage mode was divided into CSM and NCSM. We performed a meta-analysis of studies to calculate the proportion of patients with CSM after NAC grouped by hormonal receptor. Studies were considered eligible if they reported the shrinkage mode after NAChormonal receptor. hormonal receptorThe shrinkage mode was evaluated on the basis of MRI or pathology. The hormonal receptor was characterized with traditional markers [hormone receptor (ER/PR, and HER2 status)].

### Eligibility Criteria

Studies conducted with human subjects were included if they met all of the following criteria: All cases were definitely diagnosed as breast cancer and distant metastasis was excluded; cases were received neoadjuvant treatment; detailed statistics had to be reported (i.e. patient numbers and percentage of CSM based on MRI or pathological assessment); the language is Chinese or English. Any investigations that did not meet all inclusion criteria or is less than 12 points in the quality of the research publication were excluded. If data were duplicated in more than one paper, the most recent paper was included in the analysis.

### Systematic Search

We systematically searched the literature, published from January 2002 to June 2020 on shrinkage mode after NAC, from PubMed, Web of Science, Embase, The Cochrane Library, CNKI, Wanfang Database. Search terms included controlled terms (MeSH in PubMed), as well as free text terms. Search terms (“breast cancer OR breast neoplasm”) were used in combination with (“neoadjuvant OR NAC”) AND (“shrinkage OR regression”). According to the inclusion and exclusion criteria, all the literatures we obtained were non-randomized controlled studies.

### Data Extraction

Data were independently extracted by two authors (Chun-hui Zheng and Kai Xu) using the same standardized table; any disagreements were resolved by discussion and arbitrated by a third author (Yong Sheng Wang). The content of the extracted data includes the first author, the year of publication, the median age, the total number of samples, the number of CSM, the number of HR+, the number of HR+ CSM, the number of HR-patients, the number of HR-CSM, and the number of CSM in each subgroup.

### Article Quality Evaluation

MINORS entry (methodological index for non-randomized studies, MINORS) bias risk evaluation criteria was used to evaluate the included articles. The quality of the article was evaluated from eight aspects: research purpose, patient continuity, data collection, rationality of endpoint indicators, objectivity of endpoint evaluation, follow-up time, loss to follow-up rate, and sample size. The items are scored 0 (not reported), 1 (reported but inadequate) or 2 (reported and adequate). The total score is 0-16 points. When the score is ≥12 points, the risk of bias is considered to be low (7).

### Sensitivity and Publication Bias

The publication bias of the study was evaluated by funnel chart and Egger’s test. Meanwhile, funnel plots were used to visualize the potential publication bias (8). Sensitivity analysis was evaluated by being excluded one by one.

### Statistical Analysis

Stata 15.1 software was used for Meta-analysis. Homogeneity test (Q test) and I^2^ value was used to test the heterogeneity of the included research. When the heterogeneity test result is P>0.1 and I^2^<50%, the heterogeneity of the research is considered to be acceptable, and then the fixed effects model is adopted; otherwise the random effects model is adopted (9). (1) A random effects model was used to combine the included studies on the rate of CSM after NAC, calculate the rate of CSM and 95% CI, perform the sensitivity analysis with one-by-one elimination, and determine the publication bias of the study by using the funnel plot and Egger’s test (8). (2) The random effects model was used to calculate the CSM rate and 95% CI of HR+ and HR- type respectively, and the random effects model was also used to calculate the OR value of the CSM rate of HR- and HR+ type with HR+ type as the control. (3) The subtype classification was divided into four types: luminal A, luminal B, HER2 positive, and triple-negative, and the combined CSM rate was calculated separately; in spss25.0, chi-square test was used to test whether there was a discrepancy in the CSM rates of the four subtypes; Chi-square segmentation was used to compare rates of the four subtypes.

## Results

### Preliminary Screening of Articles

**Figure 1:**
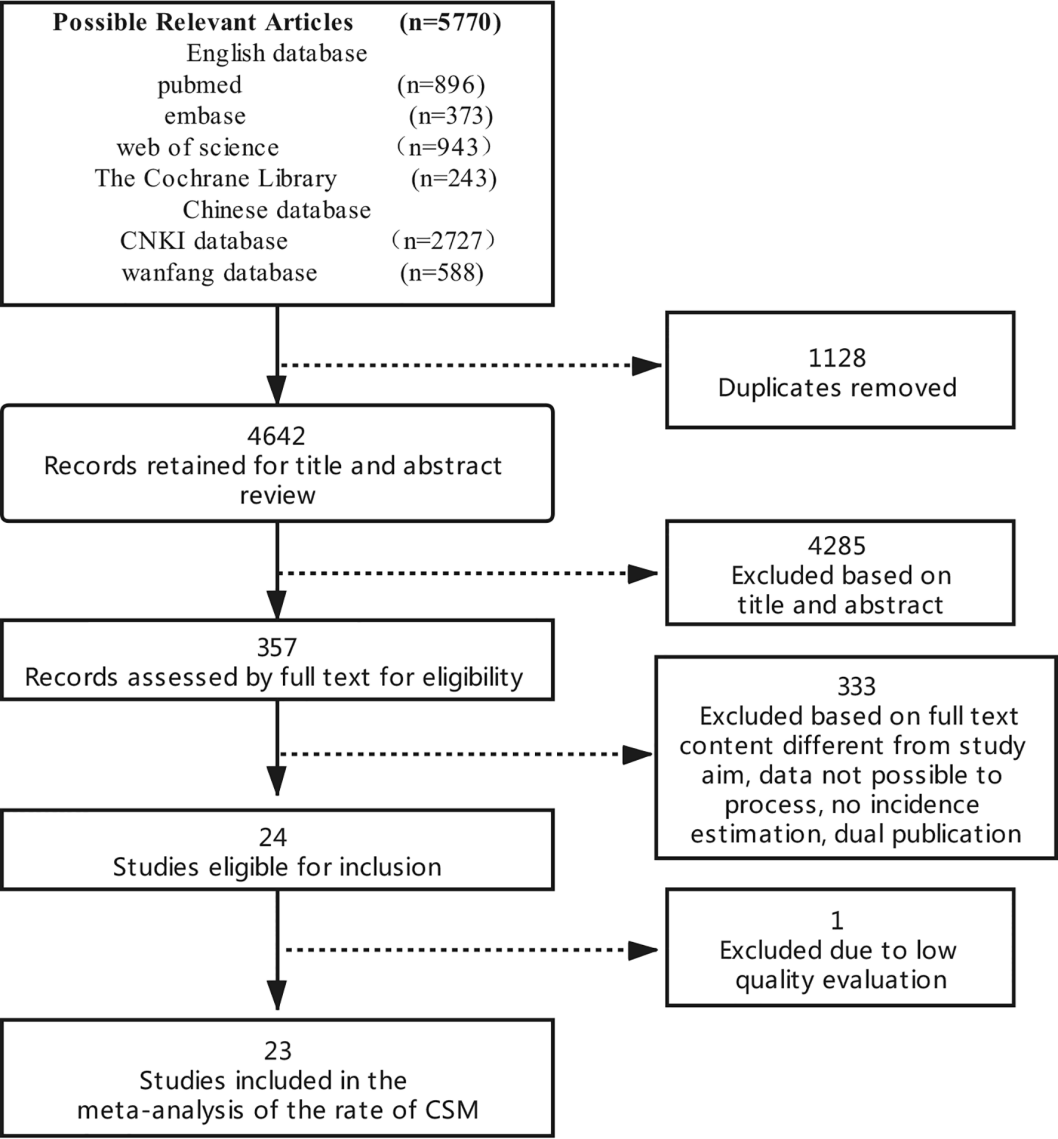
Flow chart of literature screening. CSM, concentric shrinkage mode.

Through the preliminary computer search, a total of 5770 documents were retrieved. The authors screened documents by deleting duplicate documents, reading questions, abstracts and full texts. Twenty-three documents that met standards were initially included. The specific process is shown in **Figure 1**.

### Results of the Quality Evaluation of the Articles

The MINORS quality evaluation standard was used to evaluate the quality of the initially included 24 articles. Among them, 23 articles (10–32) had quality score ≥12 points, and relatively low risk of bias, so they were included in the analysis. Only 11 of the included articles studied breast cancer hormonal receptor, A total of 2434 tumors were included, in which 1355 tumors showing CSM after NAC. Among the paper that analyzed breast cancer hormonal receptor:759 luminal tumors were included in 10 related studies; 153 HER2+ tumors were included in 8 related studies; 255 triple negative tumors were included in 9 related studies; 48 triple positive tumors were included in 2 related studies. The basic characteristics and quality evaluation results of the included studies are shown in **Table 1**.

**Table 1:** Univariable analysis and multivariable analysis of the model and clinical features.

Author	Year	List 1	List 2	List 3	List 4	List 5	List 6	List 7	List 8	MINORS Score
Nakamura, S.	2002	**1**	**0**	**2**	**2**	**1**	**2**	**2**	**0**	10(excluded)
Kim, H. J (10)	2007	**2**	**2**	**2**	**2**	**1**	**2**	**2**	**0**	13
Loo, C. E (11)	2011	**2**	**2**	**2**	**2**	**2**	**2**	**2**	**0**	14
Kim, T. H (12)	2012	**2**	**2**	**2**	**2**	**2**	**2**	**2**	**0**	14
Hu, J.X (13)	2012	**2**	**2**	**2**	**2**	**0**	**2**	**2**	**0**	12
Mukhtar, R. A (14)	2013	**2**	**0**	**2**	**2**	**2**	**2**	**2**	**0**	12
Tomida, K (15)	2014	**2**	**2**	**2**	**2**	**1**	**2**	**2**	**0**	13
Hu, Y (16)	2014	**2**	**2**	**2**	**2**	**0**	**2**	**2**	**0**	12
Xiao, R (17)	2014	**2**	**2**	**2**	**2**	**1**	**2**	**2**	**0**	13
Zhou, Q (18)	2014	**2**	**2**	**2**	**1**	**2**	**2**	**1**	**0**	12
Bansal, G. J (19)	2016	**2**	**2**	**2**	**2**	**2**	**2**	**2**	**0**	14
Liao, C. W (20)	2016	**2**	**2**	**2**	**2**	**0**	**2**	**2**	**0**	12
Ballesio, L (21)	2017	**2**	**2**	**2**	**2**	**2**	**2**	**2**	**0**	14
Eom, H. J (22)	2017	**2**	**2**	**2**	**2**	**2**	**2**	**0**	**0**	12
Li, M (23)	2017	**2**	**2**	**2**	**2**	**2**	**2**	**2**	**0**	14
Fukada, I (24)	2018	**2**	**2**	**2**	**2**	**2**	**2**	**2**	**0**	14
Goorts, B (25)	2018	**2**	**2**	**2**	**2**	**2**	**2**	**2**	**0**	14
Shin, S. U (26)	2018	**2**	**2**	**2**	**2**	**2**	**2**	**2**	**0**	14
Zhang, D (27)	2018	**2**	**2**	**2**	**2**	**2**	**2**	**2**	**0**	14
Shao, Z. Z (28)	2018	**2**	**2**	**2**	**2**	**1**	**2**	**2**	**0**	13
Xu, C.J (29)	2018	**2**	**2**	**2**	**2**	**1**	**2**	**1**	**0**	12
Ling, D. C (30)	2019	**2**	**2**	**2**	**2**	**0**	**2**	**2**	**0**	12
Liu, D.Z (31)	2019	**2**	**2**	**2**	**2**	**2**	**2**	**2**	**0**	14
Zhang, Q.C (32)	2019	**2**	**2**	**2**	**2**	**0**	**2**	**2**	**0**	12

MINORS: Methodological index for non-randomized studies; List 1: A clearly stated aim; List 2: Inclusion of consecutive patients; List 3: Prospective collection of data; List 4: Endpoints appropriate to the aim of the study; List 5: Unbiased assessment of the study endpoint; List 6: Follow-up period appropriate to the aim of the study; List 7: Loss to follow up less than 5%; List 8: Prospective calculation of the study size.

### Meta-Analysis Results

#### Estimates Rates of CSM

**Figure 2:**
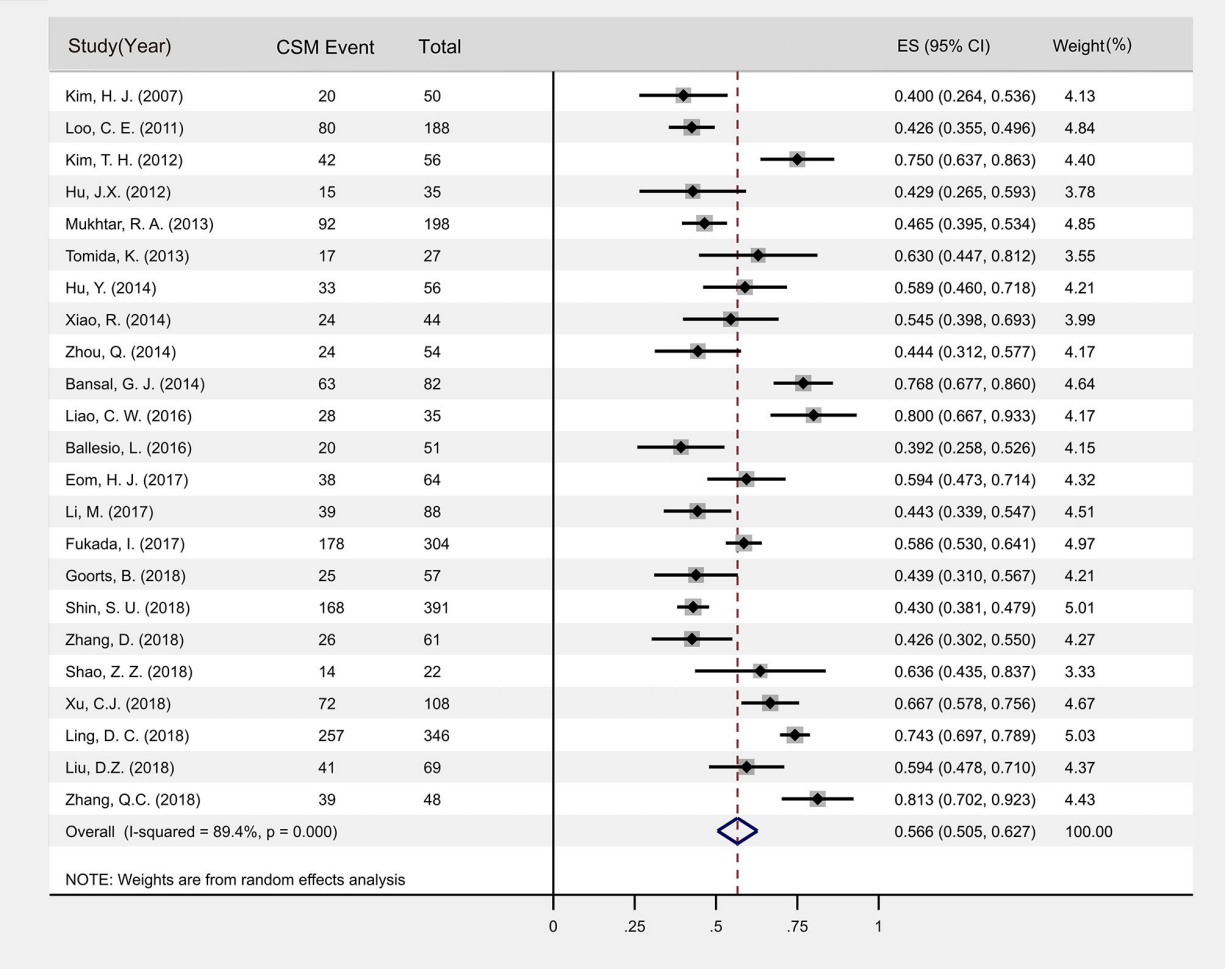
Forest plot of the incidence of CSM after NAC. Pooled estimate incidence of CSM based on 23 papers providing data from 2434 tumors; CSM, concentric shrinkage mode; CI, confidence interval; ES, effect size.

**Figure 3:**
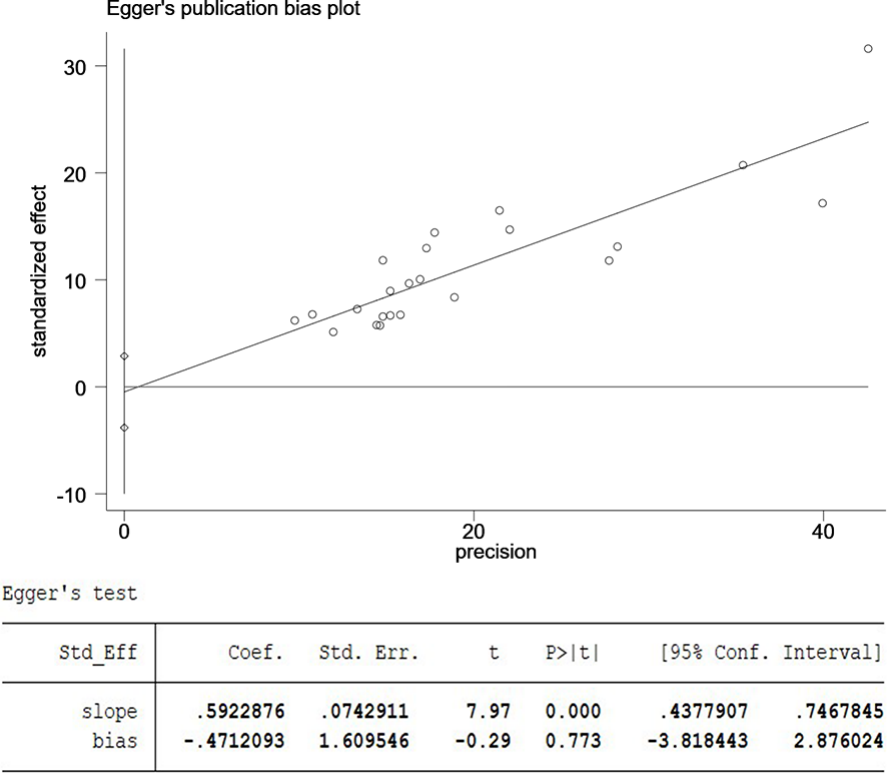
The publication bias of the study was evaluated by funnel chart and Egger’s test. CI, confidence interval; SND, standard normal deviation.

**Figure 4:**
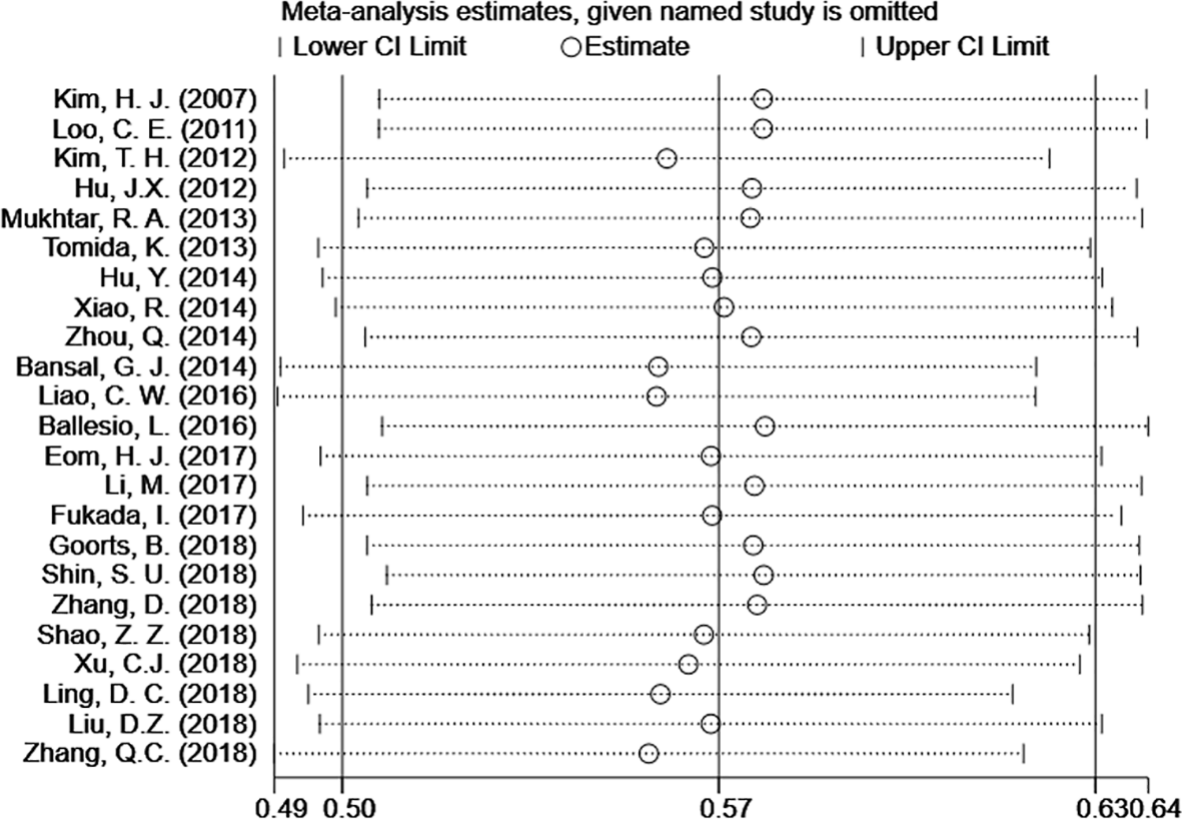
Meta-analysis estimates, given named study is omitted. The results shows that only little variation in the estimates of CSM% is induced by omission of the one selected study. CI, confidence interval.

The 23 included articles showed that the incidence of CSM after NAC was 39.2%-81.3%, **Table 2**. The results of Meta-analysis proved that there was a large heterogeneity between the included studies (I^2^ = 89.4%, P<0.001). Therefore, this study was based on the combination of the random effects model (8). The combined conversion rate based on the random effects model was 56.6% [95%CI (50.5%, 62.7%)], **Figure 2**. The publication bias of the study was evaluated by funnel chart and Egger’s test (33). publication bias, P=0.773, indicated that this article was not considered to have publication bias, **Figure 3**. Sensitivity analysis: the included 23 articles were subjected to a sensitivity analysis by being excluded one by one, indicating that each article had little influence on the combined effect size, and the results of the Meta-analysis were relatively stable, **Figure 4**.

**Table 2:** Basic characteristics and quality evaluation results of the included studies.

Study	Year	Language	Hormonal receptor	Median age	Total	CSM Events
Kim et al. (10)	2007	English	No	42 (25-68)	50	20
Loo et al. (11)	2011	English	Yes	**-**	188	80
Kim et al. (12)	2012	English	No	46 (29-63)	56	42
Hu et al. (13)	2012	Chinese	No	48.6 ± 7.2	35	15
Mukhtar et al. (14)	2013	English	Yes	48.5 (26.7–68.8)	198	92
Tomida et al. (15)	2014	English	No	-	27	17
Hu et al. (16)	2014	Chinese	No	–	56	33
Xiao (17)	2014	Chinese	No	44.12 (35.55-52.69)	44	24
Zhou (18)	2014	Chinese	Yes	–	54	24
Bansal and Santosh (19)	2016	English	Yes	47 (28-70)	82	63
Liao (20)	2016	Chinese	No	48	35	28
Ballesio et al. (21)	2017	English	Yes	No	51	20
Eom et al. (22)	2017	English	Yes	45 ± 10.09	64	38
Li et al. (23)	2017	English	Yes	46	88	39
Fukada et al. (24)	2018	English	Yes	–	304	178
Goorts et al. (25)	2018	English	No	53 (29-72)	57	25
Shin et al. (26)	2018	English	No	45.7 (22-75)	391	168
Zhang et al. (27)	2018	English	Yes	52 (39.4-64.6)	61	26
Shao et al. (28)	2018	Chinese	Yes	45	22	14
Xu et al. (29)	2018	Chinese	Yes	-	108	72
Ling et al. (30)	2019	English	No	55 (47-62)	346	257
Liu et al. (31)	2019	Chinese	No	35-72	69	41
Zhang et al. (32)	2019	Chinese	No	48	48	39

Hormonal receptor “Yes”: The articles studied breast cancer hormonal receptor.

Hormonal receptor “No”: The articles did not study breast cancer hormonal receptor.

#### Association of HR Hormonal Receptor and CSM%

**Figure 5:**
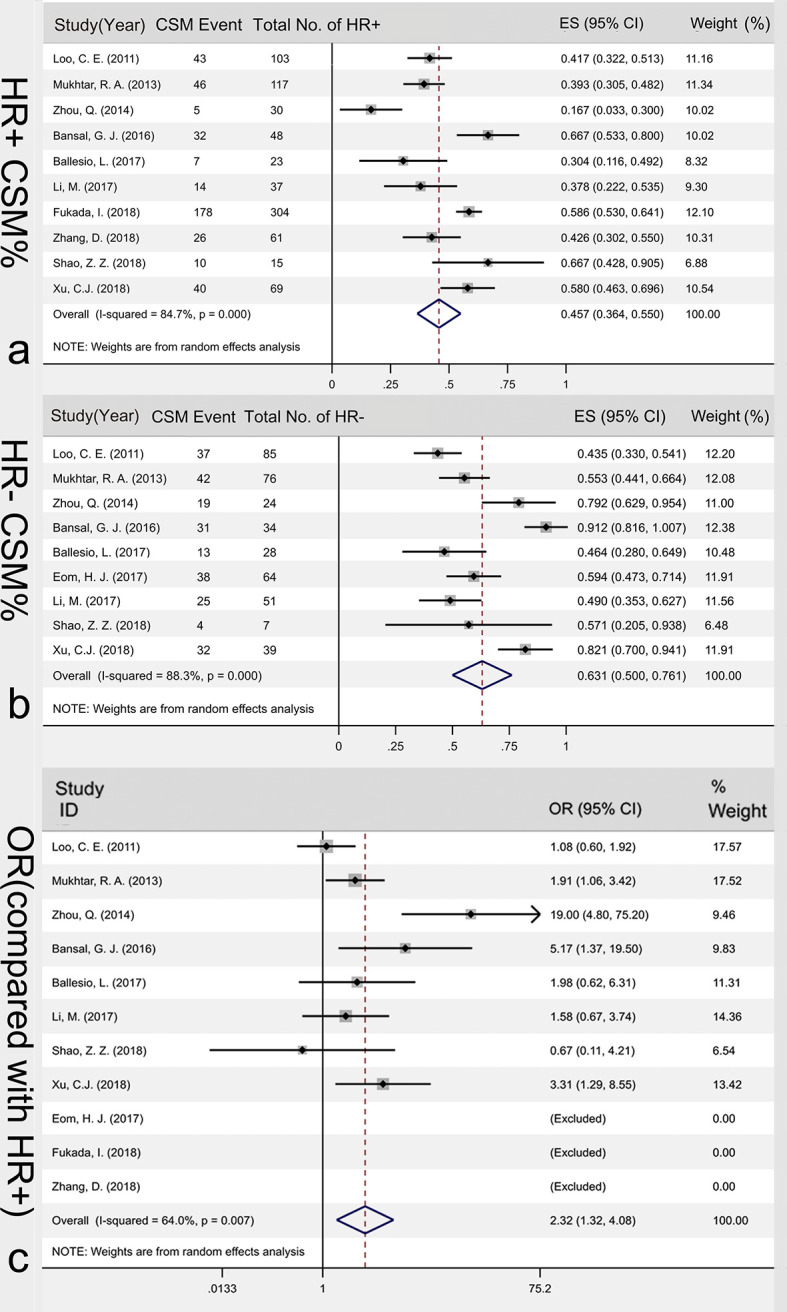
Association of HR hormonal receptor and CSM%. HR+ CSM% **(A)**, HR- CSM% **(B)** and the OR value between HR- CSM% and HR- CSM% **(C)**. CSM, concentric shrinkage mode; HR+, hormone receptor positive; HR-,hormone receptor negative; OR, odds ratio; ES, effect size.

Among the 23 articles, 10 articles analyzed HR+ type cancer, with 807 tumors included. The studies were heterogeneous (I^2^ = 84.7%, P<0.001), so random effects model was used for data combination. The results showed that the incidence of HR+ CSM was 45.7% [95%CI (36.4%, 55.0%)]. Nine articles analyzed data of HR+ cancers, with 408 tumors included. The heterogeneity of the included studies was large (I^2^ = 88.3%, P<0.001). Therefore, the random effects model was used to merge the data. The results showed that the incidence of HR- CSM was 63.1% [95%CI (50.0%, 76.1%)]. A random effects model was used to compare the CSM rates of the HR+ and HR- groups. The results of the Meta-analysis showed that the HR- patients had higher CSM rates after NAC than HR+ patients did and the OR value was 2.32 [95%CI (1.32, 4.08)], **Figure 5**.

#### Calculation of the Subtypes Specific CSM%

**Figure 6:**
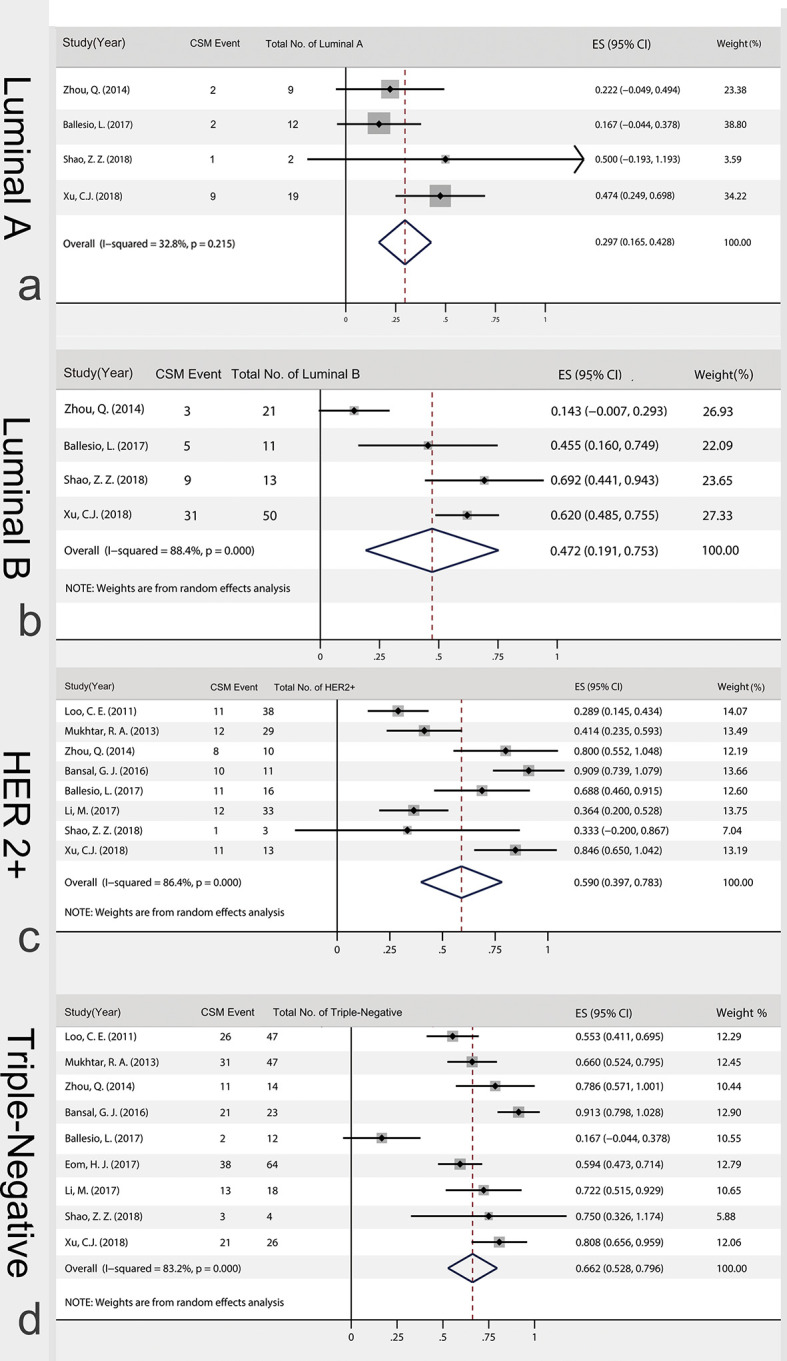
Calculation of the subtypes-specific CSM%. **(A)** 14 of 42 luminal tumors showed CSM (Luminal A); **(B)** 48 of 95 luminal B tumors showed CSM. (Luminal B); **(C)** 76 of 153 HER-2 positive tumors showed CSM (HER2+); **(D)** 166 of 255 triple-negative tumors showed CSM. (triple-negative); CSM, concentric shrinkage mode; ES, effect size.

Four articles analyzed data of luminal A breast cancer. Among 42 tumors, 14 of them showed CSM. The fixed effect model showed that luminal A CSM% was 29.7% (16.5%, 42.8%). Luminal B type was included in 4 articles. Among the 95 tumors, 48 of them showed CSM. Random effects model showed that luminal B CSM% was 47.2% (19.1%, 75.3%), **Figure 6**. Eight articles analyzed153 HER-2 positive type cases in total. The random effect model showed that the HER-2 positive type CSM rate was 59.0% (39.7%, 78.3%). Nine articles analyzed 255 triple-negative tumors in total, and the triple-negative CSM rate was 66.2% (52.8%, 79.6%). In spss25.0, chi-square test showed that the four subtypes of CSM rate had chi-square=20.932, P<0.001. The difference in CSM rate was statistically significant. It can be considered that the CSM rates of the four subtypes are unequal or not all equal. The chi-square segmentation was used to compare between the four subgroups. The results showed that the triple-negative type has the highest CSM rates, but there was no significant difference among the other three subtypes (chi-square=4.017, P=0.134).

## Discussion

Even though NAC can increase the rates of BCT in advanced breast cancer patients, the procedure remains challenging because it often results in NCSM (33). Our report analyzed a total of 23 articles, including 2434 tumors treated with NAC and evaluated for shrinkage mode. To our knowledge, this is the first study about CSM rates after NAC for breast cancers. Our data showed that 56.6% tumors treated with NAC displayed CSM. The CSM rates varies by hormonal receptor and the CSM rate of HR- patients was 2.32 times of that of HR+ patients. In subgroup analysis, we found that the triple-negative type has the highest CSM rates.

In our meta-analysis, 43.4% of breast cancer patient did not achieve CSM, potentially increasing their risk for recurrence post-BCT. It is not surprising that many scholars are cautious about increasing BCS rates considering to NAC (33, 34). With pCR rates of nearly 40% in TNBC and over 50% in HER2+ breast cancer reported, the use of NAC was continuing to increase (34). In recent years, but the proportion of NCSM might argue that NAC should be recommended for patients with suitable subsets. More measurements may be required before initial treatment to stratify the candidates who ask for BCT.

Patients with CSM can be considered to be ideal breast conserving candidates after NAC. By observing 56.6% breast cancer patients with CSM in our study, we concluded that 56.6% patients undergoing NAC can be conveyed to ideal candidates for BCT. However, the majority of NAC trials considered PCR as end points. As a result, data about rates of BCS from these studies may overestimate or underestimate the benefit of NAC for down-staging. In comparison, result from Oriana Petruolo (35), 75% (n=450) of 600 BCT-ineligible cancer patients became BCT-eligible after NAC. One hand, The discordance may be caused by the narrowly definition of CSM in our study. In fact, some patients with “limited multifocal regression” were also ideal candidates for BCT (30). In other words, we might detect a much lower rate of BCT-eligible based on CSM rates. On the other hand, the discrepancy may be influenced by the precise selection of NAC based on molecular typing in recent years. In recent clinical practice, the greatest increase in proportions of NAC was seen in TNBC and HER2 positive diseases (36), which could attribute to high CSM rates,which will be discussed in the following section. However, despite of increasing eligibility for BCS after NAC, great number of patients opted for bilateral mastectomy after NAC, even though they were fit for BCT. Indeed, in a retrospective cohort (36) study of the National Cancer Database (NCDB), only 39.7% of NAC patients underwent BCS after NAC, given that more than half of the population were BCT eligible. These findings underscored the low rates of acceptance of BCT among patients after NAC.

Hormonal receptor has important implications in clinical decision. Our results demonstrated some variability in CSM rates across different hormonal receptor. Lining up with the previous reports (35), patients with subtypes that we considered to be aggressive were ideal candidates for downs-staging with NAC. As our results show, the incidence of HR+ CSM was 45.7%; while, the incidence of HR- CSM was 63.1%. In an adjusted analysis, patients with HR negative was 2.32-folds more likely to show CSM compared to those with HR positive (odds ratio, 2.32; 95%CI, 1.32-4.08; P <.001). These findings reinforced the proposition that down-staging for BCS is a better choice for patients with HR negative cancers. However, The 45.7% CSM rates of the luminal group told us more care should be taken when patients with hormone receptor positive subtypes are selected to down-staging for BCT. This finding may explain why the reoperation rate in patients with hormone-receptor-positive are higher than patients with triple-negative or HER2-positive disease (3.5% and 6.8%, respectively; P = 0.039) (37).

We completed the analysis of subtype specific CSM rates: hormone receptor positive 45.7%, triple negative 66.2% and HER2 positive 59.0%, respectively. The result was consistent with the findings from ACOSOG Z1071: tumor biology correlates with rates of BCT. In comparison, the ACOSOG Z1071 (37) reported a real-world incidence of BCT. In their research, the rates of BCT were also higher in patients with triple-negative (46.8%) and HER2-positive tumors (43.0%) than in those with hormone-receptor-positive, HER2-negative tumors (34.5%). However, inconsistent with the highest CSM rates in triple negative tumors, it is worth noting that the HER2-positive tumors did not show the same advantage in CSM rates compared with the remaining subtypes (chi-square=4.017, P=0.134). In addition, the lower BCT rates after NAC were also reported by Surgical results from CALGB and BrighTNess trial, in which 42% to 53.2% patients with triple negative and HER2 positive completed conversion from BCT-ineligible to BCS-eligible with NAC (38–40). The discrepancy between CSM rates and BCT rates may be caused by surgeon and patient preference.

MRI was considered to be the most accurate measurement tool to estimate the residual tumor distribution after NAC, outperforming on mammography, and ultrasound (41). While, great discrepancy was reported between shrinkage mode on MRI and pathologic examination, when the shrinkage mode after NAC were accurately classified into five categories (15, 42). In fact, the accuracy of MRI was influenced by tumor morphology, histology, shrinkage pattern, and molecular subtype (43, 44). The diameter of residual tumors obtained from MRI showed a stronger agreement with residual tumor sizes, as measured using MRI and surgical specimens, in cases of concentric shrinkage mode (10, 20, 22, 32). This suggest that the CSM rates obtained based MRI appear to be reliable.

We recognized that our study had several limitations. For example, because only English and Chinese articles were included, data published in other languages were missed. Furthermore, there may also be other clinical variables, such as age, race, NAC regime, anti-HER2 which were not contained. Also, due to limited available information, we could not investigate the effects of anti-HER2 treatment on shrinkage mode in NAC. Moreover, due to limited articles with usable data, we subjectively evaluated shrinkage mode by MRI or/and pathology, given that the CSM measured by MRI was reported consistent with pathology. In addition, there was a large heterogeneity between the included articles, and the cause of heterogeneity was unknown. To combine data, random effect model was adopted, leading to a large confidence interval. Finally, since pathological assay methods were different in enrolled studies, results of our meta-analysis should be used cautiously.

## Conclusion

Although there are limitations, to our knowledge, this paper is the first to estimate the proportion of CSM after NAC. In conclusion, our meta-analysis reports that patients under NAC have an approximately 56.6% likelihood of achieve CSM. The odds of CSM for the hormone receptor negative subtypes had 2.32 times to that of the hormone receptor positive subtype. The odds of CSM were estimated to be highest for the triple negative subtypes, indicating that NAC is a desirable option for patients with these subtypes.

## Data Availability Statement

The original contributions presented in the study are included in the article/supplementary material. Further inquiries can be directed to the corresponding authors.

## Author Contributions

C-HZ and Y-SW conceived the study idea, conducted the literature searches and review of studies, performed data extraction, interpreted data analyses and drafted manuscripts. KX and C-PW performed statistical analyses and advised on data interpretation, advised on methodologies and helped draft the manuscript. Y-KZ, and Z-DS advised on clinical content and data resolution, and helped draft the manuscript. W-PS, X-JG, Y-JW, and J-YQ created the data extraction forms. All authors interpreted data and wrote the report. C-HZ and KX contributed equally to this work. All authors contributed to the article and approved the submitted version.

## Funding

This work was supported by Medical and Health Science Technology Development Program in Shandong Province (2019WS254).

## Conflict of Interest

The authors declare that the research was conducted in the absence of any commercial or financial relationships that could be construed as a potential conflict of interest.

## Publisher’s Note

All claims expressed in this article are solely those of the authors and do not necessarily represent those of their affiliated organizations, or those of the publisher, the editors and the reviewers. Any product that may be evaluated in this article, or claim that may be made by its manufacturer, is not guaranteed or endorsed by the publisher.
